# Comparative analysis of MVD and RHZ in the treatment of primary glossopharyngeal neuralgia: A clinical report on 61 cases

**DOI:** 10.3389/fneur.2023.1024142

**Published:** 2023-02-13

**Authors:** Leibo Wang, Qingjun Liu, Xiaoxia Dong, Junwei Wang

**Affiliations:** Department of Neurosurgery, Tianjin Huanhu Hospital, Tianjin, China

**Keywords:** glossopharyngeal neuralgia, MVD, RHZ, complication, efficacy

## Abstract

**Objective:**

Clinical data on 61 patients (grouped by their treatment with MVD or RHZ) with glossopharyngeal neuralgia were analyzed retrospectively. A summary analysis of the effective rate and surgical complications of MVD and RHZ in the treatment of glossopharyngeal neuralgia was performed to observe the new surgical options for GN.

**Method:**

From March 2013 to March 2020, 63 patients with GN were admitted to our hospital by the professional group of cranial nerve diseases. Two patients diagnosed with tongue and pharynx pain secondary to tongue cancer and upper esophageal cancer, respectively were excluded from the group. The remaining patients all met the diagnosis of GN, some of them were treated with MVD and others were treated with RHZ. The pain relief rate, long-term results, and complications of the patients in the two groups were well-organized and analyzed.

**Result:**

Of the 61 patients, 39 were treated with MVD and 22 were treated with RHZ. In the early-stage patients (the first 23 patients), all of them were operated on with the MVD procedure except one patient without vascular compression. In the later-stage patients, MVD was performed for evident single arterial compression according to the intraoperative situation. And for compression of arteries with greater tension or PICA + VA complex compression, RHZ was performed. It was also performed in cases where vessels with tight adhesions to the arachnoid and nerves could not be easily separated, or where it was easy to damage the perforating arteries after separating the blood vessels, causing vasospasm, which affects the blood supply to the brainstem and cerebellum. RHZ was also performed if there was no clear vascular compression. The efficiency of both groups was 100%. In the MVD group, one case recurred 4 years after the initial operation, and RHZ was performed for reoperation. Complications related to the operation included one case of swallowing and coughing in the MVD group, and three cases in the RHZ group; two cases of uvula not centering in the MVD group, and five cases in the RHZ group. There was 2 patients in RHZ group lost taste in 2/3 of the backing of the tongue, though these symptoms mostly disappeared or decreased after follow-up. One patient in the RHZ group had developed tachycardia by the time of the long-term follow-up, but whether it was related to the surgery is still uncertain. In terms of serious complications, there were two cases of postoperative bleeding in the MVD group. Based on the clinical characteristics of the patients' bleeding, it was judged that the cause of the bleeding was ischemia and was related to an intraoperative injury to the penetrating artery of the PICA artery and vasospasm.

**Conclusion:**

MVD and RHZ are effective methods for the treatment of primary glossopharyngeal neuralgia. MVD is recommended for cases where vascular compression is clear and easy to handle. However, for cases with complex vascular compression, tight vascular adhesions, difficult separation, and without clear vascular compression, RHZ could be performed. Its efficiency is equivalent to MVD, and there is no significant increase in complications such as cranial nerve disorders. There are few cranial nerve complications that seriously affect the quality of life of patients. RHZ helps to reduce the risk of ischemia and bleeding during surgery by reducing the risk of arterial spasms and injury to the penetrating arteries by separating the vessels due to separation of vessels during MVD. At the same time, it may reduce the postoperative recurrence rate.

## Introduction

Glossopharyngeal neuralgia (GN) accounts for ~0.2%−3% of all cranial neuralgia (1–4). The main clinical feature of GN is severe episodic pain in the distribution area of the glossopharyngeal nerve. The patient's pain has as a sudden onset and an abrupt stop, can be induced by swallowing, speaking, and sneezing (5), and can last from a few seconds to a few minutes (though the duration of attacks may be prolonged in some patients with a longer course of the disease or after multiple treatments (6)). Treatment for GN includes oral medication (mainly carbamazepine, oxcarbazepine, gabapentin, etc.) (7, 8), radiofrequency thermocoagulation (9), gamma knife (10, 11), and neurosurgery. Microsurgery mainly includes microvascular decompression (MVD) and rhizotomy (RHZ; here rhizotomy includes the first filament of the glossopharyngeal nerve and vagus nerve). This paper describes 61 cases of GN treated by microsurgery in our hospital. The authors conducted detailed data summaries and close follow-ups, and described them objectively, with some results differing from those previously reported in the literature.

## Materials and methods

**Figure 1:**
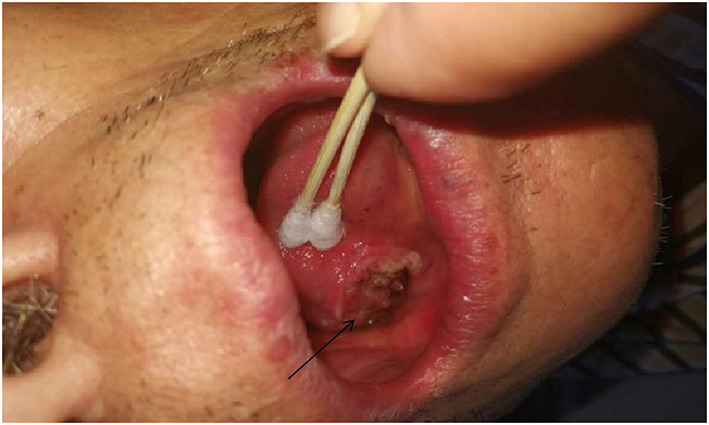
Glossopharyngeal neuralgia-like symptoms secondary to tongue cancer.

**Figure 2:**
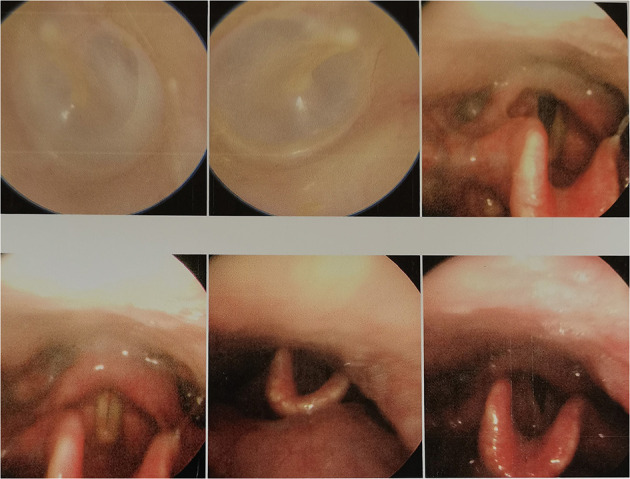
No obvious abnormality was found in patient No# through laryngoscopy. The lymphoid tissue of the root of the tongue is hyperplasia and hypertrophy, the dimple is slightly congested, and no new creatures are found.

**Figure 3:**
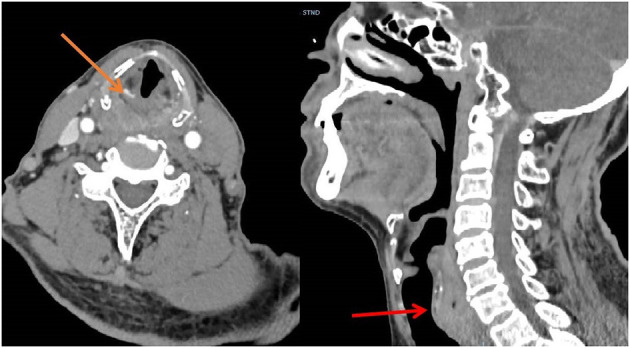
CT showed upper esophageal cancer with diffuse thickening and calcification.

From March 2013 to March 2020, There were 63 patients with pain in their tongues and throats were admitted to our department (Table 1). The symptoms of patient ^*^ were very similar to those of GN, characterized by severe pain in the right pharynx and tongue root for 3 months. The pain of patients treated with carbamazepine can be partially alleviated. Before admission, the patient had undergone laryngoscopy and CT examination of the nasopharynx and no abnormality was found. When the head MRI showed that there were blood vessels around the right glossopharyngeal nerve, the patient was admitted as GN. After the MVD of the right glossopharyngeal nerve, the pain of the patient was reduced only for 1 day, and the patient could only continue to be given carbamazepine, oxycodone, pregabalin, and clonazepam to alleviate the pain. One month later, the patient found sublingual ulceration and was diagnosed with tongue cancer by pathology (Figure 1). Patients marked with # was admitted to hospital with episodic pain in the right pharynx for 4 months, which could be induced by a swallowing action and could last from a few seconds to a few minutes. The attacks could be partially relieved by treatment with carbamazepine. The laryngoscopy did not show any significant abnormality (Figure 2). The patient reported mild choking, coughing when swallowing, and mild hoarseness after admission. As his medical history was short, the patient was sent for a CT examination of the nasopharynx and was diagnosed with upper esophageal cancer (see Figure 3). Afterward, he was diagnosed with esophageal adenocarcinoma by pathology. Following two courses of radiotherapy, the pain was significantly reduced.

**Table 1:** Information of 63 patients.

**NO**	**Gender**	**Age**	**Site**	**Pain location**	**Inducing factors**	**Preoperative course**	**Carbamazepine effect**	**Surgical**	**Other treatments**	**Compressed vessels**	**Additional**
1	F	57	L	Ph, EAC	S	3 months	Partial	MVD		PICA	
2	F	62	R	RTH, EAC	SP	2 years	Total	MVD		PICA	
3	F	63	R	Ph	S	6 months	Total	MVD		PICA	
4	M	60	L	Ph, EAC	S	15 days	Total	MVD		VA + PICA	
5	F	72	B	Ph, EAC	S	2 years	Total	MVD (L)		VA	
6	M	47	L	RTH	S, SP	1 year	Total	MVD		PICA	
7	M	56	R	RTH, Ph, A	S, SN	3 months	Ineffective	MVD		PICA	
8	F	62	L	M	S	6 months	Ineffective	MVD + TN		AICA	
9	M	46	L	RTH	S, SP	20 days	Ineffective	MVD		PICA	Cerebrospinal fluid rhinorrhea occurred after operation and was cured after lying in bed for 7 days. Pain recurrence after 46 months
10	M	49	R	RTH, EAC	S, SP	2 months	Total	MVD		VA	CS
11	F	73	L	Ph	S	7 years	Total	RHZ	RF slow down for 1 year	None	
12	M	62	L	Ph, EAC	S	6 months	Total	MVD		PICA	
13	M	43	R	Ph, M	S, SP	4 years	Total	MVD + TN		VA	
14	M	71	L	Ph, EAC		15 days	Ineffective	MVD	RF for TN ineffective	VA + PICA	Misdiagnosed as TN hemorrhage
15	M	88	L	Ph	S	2 months	Ineffective	MVD		PICA	Misdiagnosed as pharyngitis
16	F	64	L	RTH	S	5 years	Total	MVD		PICA	Misdiagnosed as pharyngitis
17	F	62	R	RTH, Ph, A, M	S, SN	19 days	Ineffective	MVD + TN		PICA	Misdiagnosed as pharyngitis
18	F	60	L	RTH, M	S	1 month	Partial	MVD		PICA	
19	M	70	R	Ph, EAC	S, chew	2 years	Total	MVD		VA + PICA	Misdiagnosed as pharyngitis
20	F	60	L	M	S, SP	15 days	Effective at first	MVD + TN		AICA	Misdiagnosed as pharyngitis
21	F	67	L	RTH, M	S, SP	13 years	Partial	MVD		PICA	Misdiagnosed as styloid process syndrome
*	M	72	R	Ph, IA	S, SP	3 months		MVD			Tongue cancer
22	F	62	R	RTH, EAC	S	26 days	Ineffective	MVD	RF ineffective	PICA	
23	F	62	L	RTH, M		5 years	Total	MVD	RF ease	VA+PICA	Hemorrhage
24	M	64	R	Ph, M	S	5 months	Ineffective	MVD		PICA	
25	M	61	L	Ph, EAC	S, SP	4 years	Total	RHZ		PICA	
26	M	61	R	EAC	S	3 years	Total	RHZ		VA + PICA	Misdiagnosed as TN
27	M	55	R	Ph, RTH	SP	15 years	Total	MVD		PICA	
28	F	60	L	Ph	S	4 years	Ineffective	MVD	RF slow down for 1 year	PICA	
29	F	63	L	Ph, EAC	S	1 month	Ineffective	MVD		PICA	CS
30	M	62	L	Ph	S	18 years	Total	MVD		VA	
31	M	59	R	RTH, EAC	S	12 years	Total	RHZ + TN		VA+PICA	
32	M	58	L	EAC	S, SP	1 month	Ineffective	MVD		PICA	
33	F	53	R	RTH, Ph, A	S	6 years	Total	RHZ	RF ease	PICA	
34	F	76	R	RTH, Ph, A, M	SP	10 years	Effective at first	RHZ	RF, MVD ineffective	None	
35	F	49	R	RTH	S	7 years	Total	RHZ		PICA	
36	M	71	L	M, EAC	S	13 years	Effective at first	MVD		PICA	
37	M	67	R	EAC	S	5 years	Total	RHZ+TN		VA+PICA	
38	M	75	L	RTH, M	S	6 months	Ineffective	MVD		PICA	
39	F	58	R	EAC	S, SP	10 years	Total	RHZ (untreated vagus nerve)		PICA	
40	M	64	L	RTH, M	S, SP	4 years	Effective at first	MVD		VA	
41	F	76	L	RTH, EAC	S	4 years	Effective at first	RHZ		AICA	
42	F	68	R	Ph, EAC	S	9 years	Effective at first	MVD		PICA	
43	M	64	L	RTH	S, SN	4 years	Total	RHZ		PICA	Misdiagnosed as pharyngitis
44	F	88	R	EAC	S	11 days	Ineffective	MVD		PICA	
45	M	65	L	M	S	10 years	Effective at first	RHZ + TN		AICA	
46	M	52	R	EAC	S, SP	2 years	Total	RHZ		VA	CS
47	F	72	R	RTH	S	4 years	Total	RHZ		VA+PICA	
48	F	56	L	RTH, M	S	4 years	Total	MVD		PICA	
49	M	50	L	Ph, IA	S, SP	7 days	Ineffective	RHZ		PICA	No. 9 are the same patient
50	M	58	L	RTH	S, SN	10 years	Total	MVD		PICA	
51	M	68	L	Ph, EAC	S	2 years	Partial	MVD		PICA	Misdiagnosed as pharyngitis
#	M	68	L	Ph, RTH	S, chew	3 months					Carcinoma of upper esophagus
52	M	67	L	RTH	S	1 year	Total	RHZ		PICA	Misdiagnosed as pharyngitis
53	M	72	R	Ph	S, SP	2 years	Total	MVD		PICA	Misdiagnosed as TN
54	M	60	L	Ph	S	17 months	Ineffective	MVD		PICA	
55	F	65	L	Ph	S, SN	3 months	Total	MVD		PICA	Misdiagnosed as TN
56	F	67	L	Ph	S	2 years	Total	RHZ		PICA	
57	M	72	L	RTH, EAC	S, SN, SP	2 months	Total	RHZ		PICA	
58	F	56	R	Ph	S	5 years	Total	RHZ		VA	Misdiagnosed as pharyngitis
59	F	63	R	Ph	S	6 months	Total	RHZ		PICA	
60	F	46	R	Ph, EAC	S, SP	2 years	Total	RHZ	RF ineffective	PICA	Misdiagnosed as styloid process syndrome
61	M	67	R	Ph	S, chew	1 months	Ineffective	RHZ	RF ineffective	VA	

L, left; R, right; M, male; F, female; Ph, pharynx; EAC, external auditory canal; RTH, root of the tongue; M, mandible; A, auricle; IA, inferior alveolar; S, swallow; SP, speak; SN, sneeze; RF, radio frequency; CS, cardiovascular symptoms.

As the two patients mentioned above belonged to secondary GN, they were excluded from this study. The remaining 61 patients were in line with ICDH3 (7) (Table 2).

**Table 2:** Diagnostic criteria of glossopharyngeal neuralgia.

A. Recurring paroxysmal attacks of unilateral pain in the distribution of the glossopharyngeal nerve1 and fulfilling criterion B
B. Pain has all of the following characteristics:
1. Lasting from a few seconds to 2 min
2. Severe intensity
3. Electric shock-like, shooting, stabbing, or sharp in quality
4. Induced by swallowing, coughing, talking, or yawning
C. Not better accounted for by another ICHD-3 diagnosis
Within the posterior part of the tongue, tonsillar fossa, pharynx, or angle of the lower jaw and/or in the ear

The youngest patient in this group was 43 years old and the oldest was 88 years old. The mean age was 67.72. Thirty-two patients were male and 29 were female. There were 35 cases of left-sided pain, 25 cases of right-sided pain, and one case of bilateral pain. In terms of the area of pain, all patients felt pain at the base of the tongue or in the pharynx. Twenty-four (39.3%) patients felt pain in the external auditory canal, of which only 6 (9.8%) patients had pain in the external auditory canal alone.

**Figure 4:**
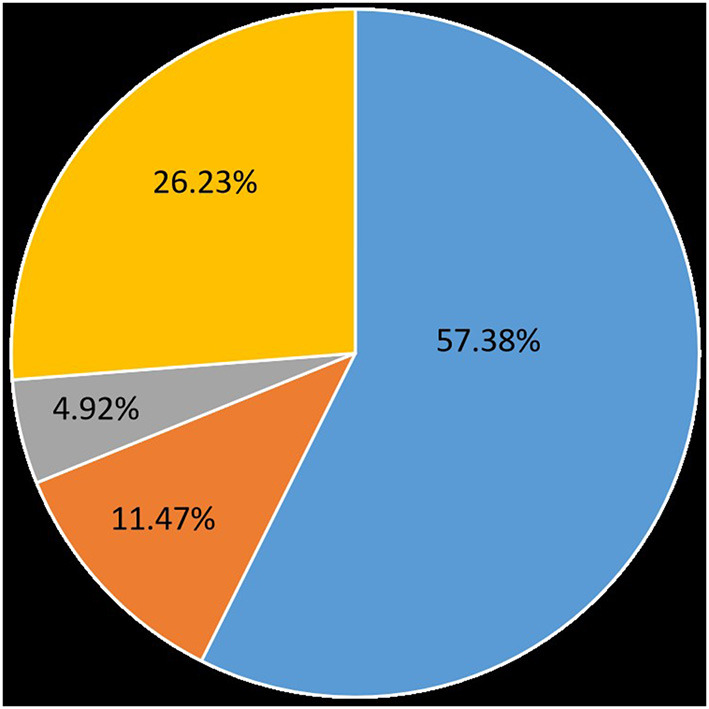
Effectiveness of carbamazepine on patients with glossopharyngeal neuralgia. 57.38% were on complete remission, 4.92% were on partial remission, early-stage effective accounted for 11.47%, 26.23% took carbamazepine without remission.

In this group, oral carbamazepine was used in the early stage. Forty-five patients were on carbamazepine in remission, 35 (57.38%) were in complete remission, and three (4.92%) were in partial remission (decrease in VAS pain score ≥ 3), effective in the early stage (within 3 months) in 7 patients (11.47%). Another 16 (26.23%) took carbamazepine without remission (Figure 4). The side effects of carbamazepine included dizziness and drowsiness in 12 cases, exfoliative dermatitis in three cases, and hematological suppression in 18 cases. Hematological suppression was most commonly manifested as a decrease in white blood cells, a decrease in platelets, and a decrease in fibrinogen, with hypofibrinogenemia being the most common (between 1.4 and 1.7 g/L). Some patients required fibrinogen supplementation to normal values (>2 g/L) before surgery. There were four cases of mild liver function impairment, and the patients had no conscious symptoms. Urinary incontinence was reported in one case (not yet reported). Overall, patients on whom carbamazepine was ineffective, or whose pain was only partially relieved, as well as those who could not tolerate carbamazepine, had a short preoperative course of treatment and often required urgent surgical treatment, whereas patients who had significant pain relief on carbamazepine and who tolerated carbamazepine well had a relatively long course. The average duration of preoperative disease in this group was 44.13 months, the longest was 18 years, and the shortest was 7 days.

### Treatment

**Figure 5:**
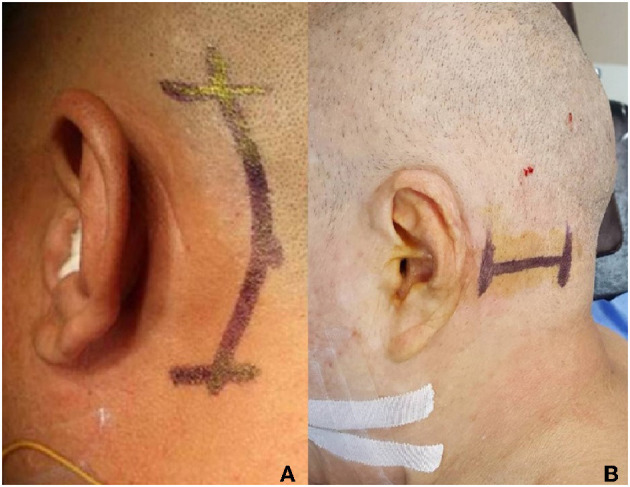
Surgical incision for glossopharyngeal neuralgia, **(A)** longitudinal incision, and **(B)** transverse incision.

Operation process: minimally invasive approach to surgery *via* retrosigmoid is adopted, with a horizontal or vertical incision (Figure 5). The surgical incision is small, with little damage to patients and fast recovery. The specific operation process will not be described in detail in this article.

All patients were examined by preoperative magnetic resonance imaging time-of-flight (MRI-TOF) brain scan. There is communication in detail before the operation, explaining that MVD or RHZ operation may be adopted according to the intraoperative conditions, and explaining the risks of the operation. The patients and their families must fully understand and approve.

**Figure 6:**
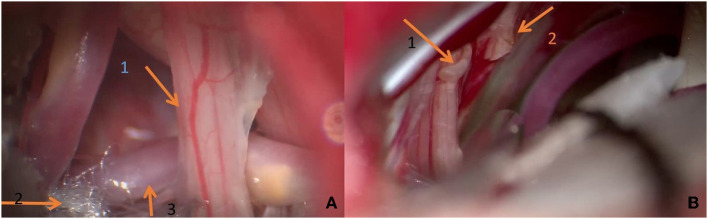
**(A)** During the operation, it can be seen that the vertebral artery compresses the glossopharyngeal nerve and forms a displacement. Teflon is being used to push it away. **(B)** Arrow 1 shows the broken end of the first branch of the vagus nerve; broken end of glossopharyngeal nerve shown by arrow 2.

Microvascular decompression was performed in the early stage. Before patient No. 23, only one patient was treated with RHZ as the exact vascular compression was not seen during the operation. The concept of operation was changed in the later stage, and MVD was generally performed for simple arterial compression. RHZ was applied to patients with high tension artery compression or PICA + VA complex compression, which made it difficult to lift up the blood vessels, risked blood vessels bouncing back to their original position, and relapsed easily. It was also performed on patients whose blood vessels tightly adhered to the arachnoid membrane and nerve and were not easy to separate, or those where it was easy to form an included angle of blood vessels after forcibly separating them, easily damaged the brain stem and cerebellar perforating arteries, easily caused vasospasm leading to brain stem ischemia, and had no obvious blood vessel compression (Figure 6). In this group, 39 patients were treated with the MVD procedure, while 21 patients were treated with the RHZ procedure. A patient (No. 39) was merely treated with lingual pharyngeal neurectomy as the adhesion between the root wires of the vagus nerve and the arachnoid membrane was extremely serious, and it was close to the brain stem, so it could not be separated and cut off. However, the patient was still classified in the RHZ group. In the two groups, because the patients' symptoms were indistinguishable from trigeminal neuralgia, trigeminal nerve exploration was performed on four and three patients, respectively. The patient information for the RHZ and MVD groups is shown in Table 3.

**Table 3:** Basic information of patients in MVD group and RHZ group.

	**MVD (*n* = 39)**	**RHZ (*n* = 22)**
Age (year)	62.87 ± 13.17	62.45 ± 12.94
**Gender**		
Male	21	11
Female	18	11
**compressed blood vessels**		
PICA	27	11
VA + PICA	4	4
VA	5	3
AICA	2	2
None	0	2
**Site**		
Left	26 (including both sides one)	10
Right	13	12

## Results

During the operation, we found that the inferior cerebellar artery (PICA) was the most common, accounting for 39 cases, with no definite vascular compression seen in two cases and vertebral artery (VA) compression in eight cases. The anterior inferior cerebellar artery (AICA) compressing in four cases, and the remaining eight cases were combined impressed by the VA + PICA complex.

**Figure 7:**
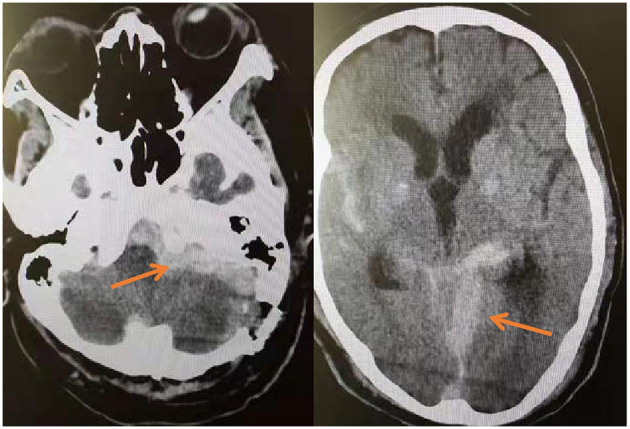
On the third day after MVD, the patient's blood pressure increased intermittently, and the corresponding antihypertensive treatment was given. On the fourth day, the patient suddenly vomited with progressive disturbance of consciousness. The reexamination of head CT showed the operation area and subarachnoid hemorrhage.

Postoperative pain disappeared in two groups. Two patients in the MVD group experienced postoperative hemorrhage (Figure 7), so urgently underwent emergency hematoma removal + debridement decompression. Patient No. 12 died of multi-organ failure after surgery, and patient No. 23 was treated and discharged with mild dizziness. All patients underwent cranial nerve examination of the 9th, 10th, and 11th pairs 14 days after the operation (Table 4).

**Table 4:** On the 14th day after operation, the cranial nerve functions of the IX, X and Xi groups were examined.

**Items**	**MVD (*n* = 39)**	**RHZ (*n* = 22)**
Uvula not centered	0	5
Lifting deviation of soft palate	0	0
Hoarseness of pronunciation	0	0
Swallowing cough	1	3
Disappearance of pharyngeal reflex	0	0
1/3 taste loss behind the tongue (abnormal)	0	2

The patients in this group were followed up continuously after operation. As of March 2022, the patients in this group were monitored for between 24 and 108 months. Five patients were lost to follow-up; however, all of them had been monitored for more than 24 months after surgery. The main reason they could not be monitored was death from other diseases or severe disability, or because they were unable or unwilling to participate in follow-up. In the MVD group, one patient had a recurrence 46 months after surgery, and RHZ was performed for reoperation. Patient No. 5 with bilateral glossopharyngeal neuralgia, with the left side being the most severe, and after decompression of the left glossopharyngeal nerve, the patient's pain disappeared bilaterally.

One patient with coughing and swallowing problems in the MVD group experienced partial relief 3 months later. In the RHZ group, five patients with uvula deviation improved partially after 6 months of follow-up. In two out of three patients, problems with coughing and swallowing disappeared 3 months later, and one patient's symptoms were significantly alleviated, with only occasional coughing and swallowing problems. The two patients who lost their sense of taste did not feel any confusion.

## Discussion

In 1910, Weisenburg (12) described the symptoms of glossopharyngeal neuralgia in a man of 35 who suffered from the compression of the ninth cranial nerve due to a tumor of the cerebellopontine angle (CP). In 1921, Harris (13) first named these symptoms “glossopharyngeal neuralgia.” At present, vascular compression of the root of the GN is still recognized as the most important factor by many scholars. In addition to drug therapy, the treatment of GN mainly includes CT-guided radiofrequency thermocoagulation, with an average efficiency of 78.8% (14); and gamma knife stereotactic radiotherapy, with an average efficiency of 73% (15). A craniotomy is currently considered to be the most effective treatment modality. The efficiency of microsurgery for GN has been reported to be very high in much of the previous literature (15, 16), both for MVD and RHZ.

GN was often misdiagnosed due to low incidence and clinical uncommonness especially in the early stage of the disease. Patients preferred to seek clinical help in otorhinolaryngology, and many were misdiagnosed with inflammatory lesions in the oral cavity. Nine patients in this group were misdiagnosed with pharyngitis, two patients were diagnosed with hypertelorism, and four patients were misdiagnosed with trigeminal neuralgia. In fact, misdiagnosis of glossopharyngeal neuralgia as trigeminal neuralgia was relatively common because in addition to the symptoms of the two being very similar, there was often an overlap and confusion at the site of the attack (17). In this group, seven patients experienced their pain spreading to the junctional area of the glossopharyngeal nerve and the trigeminal nerve (mandible, auricle, tongue, etc.). TN could not be excluded, and the trigeminal nerve was decompressed during operation. Other literature (16) contains similar reports.

Two patients excluded from the group were misdiagnosed with primary GN due to esophageal and tongue cancer, and pharyngeal and upper esophageal cancer also displayed a group of lesions that could be easily misdiagnosed as GN, as mentioned in Bharti et al. (9). Therefore, during the diagnosis of glossopharyngeal neuralgia, we should not only focus on the clinical performance of the patient but should also conduct more imagological examinations as far as possible. For patients whose duration of disease was short, CT examinations of nasopharynx, laryngoscopy, and intraoral probing should be given special attention. Occult tongue cancer was often not easily detected, and even early laryngoscopy and CT may not always lead to a definitive diagnosis, hence more attention should be paid to it. Blumenfeld et al. (2) also emphasizes that in addition to symptoms, the diagnosis of GN also depends on the evidence of a high-resolution CT scan and consultation with otorhinolaryngologists.

The age and lateral distribution of patients in this group are roughly consistent with the previous literature (16, 18). The effective rate of carbamazepine in this group of patients was approximately 73.77%. It was close to the effective rate of 75.9% for carbamazepine in trigeminal neuralgia (19). Only three patients in this group had symptoms of episodic arrhythmias such as bradycardia but had no serious events such as syncope. In fact, severe syncope symptoms have been rarely reported internationally; Burfield et al. (20) and Aguiar et al. (21) have reported relevant cases.

MVD was generally performed for treatment in early cases, and individual approaches were used in later cases. The use of the MVD or RHZ procedure shall be determined according to the situation of vessel compression of the patient. In the MVD group, two patients developed delayed hematoma on the second and the fourth day after operation, respectively. It should be noted that the surgeon had previously performed over 1,500 cranial nerve surgeries and had extensive clinical surgical skills and experience. There were only two cases where a late postoperative hemorrhage occurred, one case of facial spasm, and one case of trigeminal neuralgia. In this group, the cases of two patients with a postoperative hemorrhage were probably caused by vasospasm or injury to the penetrating arteries. Both patients with hemorrhages involved the PICA. The PICA had an extremely important role and position among the three arteries of the posterior circulation. The PICA artery was complementary in function to the AICA, and was particularly important because of its proximity to the brain stem, and there were multiple perforating arteries supplying blood (22). Once the spasm of the PICA or injury to the penetrating arteries happens, resulting in brainstem ischemia, it can cause dorsolateral medullary syndrome, choking on water, Horner's syndrome, vertigo, and other related symptoms. During the occurrence of the series of ischemia, patients will reflexively raise blood pressure to improve ischemia, leading to post-ischemic bleeding (23). RHZ can be considered for compression of a perforating artery that is difficult to separate and easy to damage.

Some studies also reported several patients with GPN caused by an adhesive arachnoid (24, 25). There were also reports that glossopharyngeal nerve compression arose from cerebellar choroid plexus (26). Two patients in this group had no exact vessel seen intraoperatively. The arachnoid adhesions were not severe, and the glossopharyngeal nerve was not displaced or deformed by the arachnoid retraction, and no other compressions were found after careful identification, so nerve root dissection was chosen.

The operations in the two groups were all effective; the efficiency was 100%. At follow-up, one patient in the MVD group had a recurrence 46 months after surgery and underwent RHZ surgery again. There was no recurrence in the cut-off group at the time of follow-up. Complications unrelated to the operation mode are not discussed here. In terms of complications related to the surgical approach, although the group in which nerves were cut had slightly more dysfunction than the MVD group in terms of non-centralization of the uvula and loss of taste in the posterior 1/3 of the tongue, the patients themselves did not experience any significant dysfunction. Regarding the swallowing function, one case in the MVD group and three cases in the RHZ group had swallowing and coughing problems after the operation. However, after 3 months of follow-up, only one patient in the RHZ group described having an extremely slight cough occasionally, and the other patients had no swallowing or coughing problems. One patient in the RHZ group displayed tachycardia after 1 year of follow-up. Whether this was related to the surgery was hard to determine, and there were no relevant reports in the literature. However, the glossopharyngeal nerve supplies the CB and carotid sinus (1). It conveys chemoreceptor and stretches baroreceptor information centrally for respiratory and circulatory reflex function (27). This may explain the phenomenon of heart-rate change.

For glossopharyngeal neuralgia, there have been different views on decompression or amputation. In a report on a series of 37 patients with GN, 22 underwent MVD, and 15 underwent MVD + GNR. Between the two groups, there was no significant difference in postoperative efficacy, while the incidence of postoperative hoarseness and a cough when drinking in the MVD + GNR group was higher than in the MVD group (28). In terms of efficiency, the report is close to our results, but in terms of cranial nerve complications, we believed that the cutting-off group was not particularly obvious, and many patients did not have any discomfort. Sampson et al. (29) reported an incidence of cranial nerve complications after MVD of about 11%, which was close to the incidence of the RHZ procedure.

Scranton (30) reported the outcomes of 454 patients from 14 different series undergoing microvascular decompression of cranial nerves IX and X. The rate of resolution of pain for patients undergoing MVD was 84.7% with recurrence in 7% of patients. Transient cranial nerve X dysfunction occurred in 13.2% of patients and a permanent cranial nerve deficit occurred in 5.5%. In contrast, the long-term rate of pain control for 157 patients who underwent RHZ was higher at 87.3%; however, the rate of transient and permanent cranial nerve deficits was increased to 25%, and 19.1%, respectively. Rui (31) et al. reviewed 37 patients with GN, of whom 22 underwent MVD and 15 underwent MVD + RHZ, with no statistical difference in efficiency between the two groups, while postoperative complications were slightly higher in the latter than in the former. We believed that there was no difference in pain relief between the two group and that RHZ was only slightly higher than MVD in terms of transient cranial nerve dysfunction, and patients have nonsense for most of this neurological dysfunction. In fact, we found that after one side of the glossopharyngeal nerve was severed, the patient had no obvious symptoms. On the contrary, if the glossopharyngeal nerve was not completely severed, but only injured or the nerve nucleus damaged, it would produce obvious swallowing and choking symptoms. The above reasons are not clear. We consider that they mainly come from the following points: because the oropharyngeal space is extremely small, water or food will trigger bilateral information effectors at the same time. Even if one side of the glossopharyngeal nerve is cut off, complete information can still be transmitted to the advanced center through the opposite side of the normal glossopharyngeal nerve. The center will start bilateral efferent impulses synchronously, and because the bilateral hypoglossal nerves and vagus nerves are consistent, dysphagia and cough will not occur. However, when one side of the glossopharyngeal nerve is only partially damaged, due to the inconsistency of bilateral information transmission the movement of both sides of the tongue and pharyngeal muscles will be uncoordinated, resulting in a series of symptoms.

RHZ was safer in terms of surgical risk compared with MVD, which included the risk of injury to the penetrating artery, vasospasm, and angulation. These risks may lead to more serious short-term life-threatening complications such as postoperative ischemia and bleeding. Compared with cranial nerve dysfunction, more attention should be paid to risk reduction.

## Limitations

Limitations of this study include the retrospective nature of the analysis (although most surgical GN literature is retrospective). Patients were not randomly assigned to a procedure, and there was no blinding of the procedure to clinicians or patients. Indeed, there is a major need for more randomized controlled trials in the neurosurgical treatment of GN, though randomization poses its own challenges. Compared with previous literature, this study showed that RHZ did not significantly increase the patient's extension dysfunction. As for the difference in recurrence rate, a longer follow-up period is needed.

## Conclusion

We believe that both MVD and RHZ are very effective in treating GN. RHZ did not increase the patient's cranial nerve dysfunction excessively. There was no need to be too concerned about whether there would be neurological dysfunction, but more attention should be paid to the situation of vascular compression, and whether it is easier to separate intraoperatively. In order to reduce the potential risk of delayed postoperative ischemia and hemorrhage as much as possible, we suggest that either MVD or RHZ should be carried out according to the patient's intraoperative compression to reduce the complication.

## Data availability statement

The original contributions presented in the study are included in the article/supplementary material, further inquiries can be directed to the corresponding author.

## Ethics statement

Ethical review and approval was not required for the study on human participants in accordance with the local legislation and institutional requirements. Written informed consent from the patients/participants or patients/participants' legal guardian/next of kin was not required to participate in this study in accordance with the national legislation and the institutional requirements.

## Author contributions

LW: responsible for the overall design, data collection, and writing of the article. XD: patient follow-up and communication. JW: responsible for statistical work and article translation. QL: responsible for clinical related work. All authors contributed to the article and approved the submitted version.
